# Benchmarking AI scientists for omics data–driven biological discovery

**DOI:** 10.1093/bioinformatics/btag227

**Published:** 2026-07-07

**Authors:** Erpai Luo, Jinmeng Jia, Yifan Xiong, Xiangyu Li, Xiaobo Guo, Baoqi Yu, Minsheng Hao, Lei Wei, Xuegong Zhang

**Affiliations:** MOE Key Laboratory of Bioinformatics and Bioinformatics Division of BNRIST, Department of Automation, Tsinghua University, Beijing, 100084, China; MOE Key Laboratory of Bioinformatics and Bioinformatics Division of BNRIST, Department of Automation, Tsinghua University, Beijing, 100084, China; MOE Key Laboratory of Bioinformatics and Bioinformatics Division of BNRIST, Department of Automation, Tsinghua University, Beijing, 100084, China; School of Software Engineering, Beijing Jiaotong University, Beijing, 100044, China; Department of Physiology and Pathophysiology, School of Basic Medical Sciences, Capital Medical University, Beijing, 100069, China; Department of Physiology and Pathophysiology, School of Basic Medical Sciences, Capital Medical University, Beijing, 100069, China; MOE Key Laboratory of Bioinformatics and Bioinformatics Division of BNRIST, Department of Automation, Tsinghua University, Beijing, 100084, China; MOE Key Laboratory of Bioinformatics and Bioinformatics Division of BNRIST, Department of Automation, Tsinghua University, Beijing, 100084, China; MOE Key Laboratory of Bioinformatics and Bioinformatics Division of BNRIST, Department of Automation, Tsinghua University, Beijing, 100084, China; Center for Synthetic and Systems Biology, School of Life Sciences and School of Medicine, Tsinghua University, Beijing, 100084, China

## Abstract

**Motivation:**

Recent advances in large language models have enabled the emergence of AI scientists that aim to autonomously analyze biological data and assist scientific discovery. Despite rapid progress, it remains unclear to what extent these systems can extract meaningful biological insights from real experimental data. Existing benchmarks either evaluate reasoning in the absence of data or focus on predefined analytical outputs, failing to reflect realistic, data-driven biological research.

**Results:**

Here, we introduce BAISBench (Biological AI Scientist Benchmark), a benchmark for evaluating AI scientists on real single-cell transcriptomic datasets. BAISBench comprises two tasks: cell type annotation across 15 expert-labeled datasets, and scientific discovery through 193 multiple-choice questions derived from biological conclusions reported in 41 published single-cell studies. We evaluated several representative AI scientists using BAISBench and, to provide a human performance baseline, invited five graduate-level bioinformaticians to collectively complete the same tasks. The results show that while current AI scientists fall short of fully autonomous biological discovery, they already demonstrate substantial potential in supporting data-driven biological research. These results position BAISBench as a practical benchmark for characterizing the current capabilities and limitations of AI scientists in biological research. We expect BAISBench to serve as a practical evaluation framework for guiding the development of more capable AI scientists and for helping biologists identify AI systems that can effectively support real-world research workflows.

**Availability and implementation:**

https://github.com/EperLuo/BAISBench, https://huggingface.co/datasets/EperLuo/BaisBench.

## Introduction

Biological research increasingly relies on large-scale, data-driven analyses to elucidate cellular states, molecular mechanisms, and disease processes. Advances in high-throughput technologies have enabled the routine generation of complex datasets; however, transforming these data into biologically meaningful insight remains a labor-intensive and expertise-dependent process. From quality control and normalization to downstream interpretation and hypothesis formulation, successful analysis requires not only technical proficiency but also substantial domain expertise and biological insights. As datasets continue to grow in size and complexity, the analytical burden placed on individual researchers has emerged as a major bottleneck in modern biological research.

This challenge is especially pronounced in omics-driven studies such as transcriptomics, proteomics, and metabolomics. Among these, single-cell transcriptomics has emerged as a cornerstone technology, enabling molecular profiling at single-cell resolution and providing unprecedented access to cellular heterogeneity, rare populations, and dynamic biological processes across tissues and disease states (Zheng et al. 2017, Svensson et al. 2018). A typical single-cell RNA sequencing (scRNA-seq) analysis involves multiple stages, including data preprocessing, dimensionality reduction, clustering, and cell type annotation, followed by downstream analyses such as differential gene expression, trajectory inference, and cell–cell communication analysis. While many of these steps are supported by mature computational pipelines, researchers must still carefully select appropriate tools and parameters and integrate analytical results with prior biological knowledge to derive biologically coherent conclusions (Luecken and Theis 2019, Heumos et al. 2023). All these processes remain difficult to automate and continue to rely heavily on expert judgment.

Recent advances in large language models (LLMs) have renewed interest in intelligent systems that aim to assist or automate components of the biological research workflow. Enabled by tool use, code generation, and interaction with external knowledge sources, a new class of systems, often referred to as AI scientists, has been proposed to autonomously analyze experimental data, reason about biological phenomena, and generate candidate scientific conclusions (Lu et al. 2024a, Su et al. 2024, Gottweis et al. 2025, Yamada et al. 2025). Early studies suggest that such systems can support data analysis and hypothesis generation, raising the possibility that parts of the biological discovery process may be, at least partially, automated (Zhang et al. 2024, Reddy and Shojaee 2025).

Motivated by this vision, a growing number of AI scientist systems have been developed for biological research. For example, AutoBA (Zhou et al. 2024) utilizes an LLM agent to construct automated pipelines for preprocessing and analyzing single-cell data. scChat (Lu et al. 2024b) combines a multi-agent architecture with retrieval-augmented generation to enable autonomous exploration of single-cell datasets. BioChatter (Lobentanzer et al. 2025) provides a generic backend framework for integrating conversational AI with biomedical tools. Biomni (Huang et al. 2025) introduces a general-purpose AI scientist framework that integrates literature retrieval, tool use, and reasoning to support autonomous biomedical workflows. Pantheon enables structured scientific reasoning and iterative experiment planning through coordinated LLM agents. STELLA (Jin et al. 2025) combines LLMs with tool-assisted data analysis to facilitate data-driven scientific discovery and hypothesis validation. Collectively, these efforts highlight rapid progress in system design and integration, but they also raise a critical question: what biological capabilities do current AI scientists actually possess?

Despite growing enthusiasm, the rigorous evaluation of AI scientists in realistic biological research settings remains an open challenge. Scientific discovery is inherently open-ended, and its quality is difficult to quantify using conventional metrics. Existing evaluation strategies often rely on expert review of AI-generated conclusions (Gottweis et al. 2025, Schmidgall et al. 2025) or on LLM-based judges that approximate expert assessment (Baek et al. 2024). While informative, these approaches are subjective, difficult to scale, and provide limited insight into whether an AI system can genuinely extract biologically meaningful knowledge from raw experimental data.

Several benchmarks have attempted to address this gap by evaluating AI agents on structured scientific tasks or question-answering settings (Laurent et al. 2024, Qi et al. 2024). However, these benchmarks are primarily knowledge-driven and do not reflect the data-centric nature of biological research, where new insights emerge from the analysis of newly generated omics datasets rather than from static background information alone. The BLADE benchmark (Gu et al. 2024) partially addresses this limitation by incorporating data-driven questions, but focuses largely on domains outside molecular and cellular biology. BixBench (Mitchener et al. 2025) further grounds evaluation in real omics datasets across diverse biological scenarios, yet it mainly assesses computational outputs, without directly probing whether AI systems can produce interpretable, insight-rich biological conclusions. These limitations underscore the need for evaluation frameworks that align more closely with the core objectives of biological research: deriving biologically meaningful interpretations and discoveries from complex experimental data.

**Figure 1:**
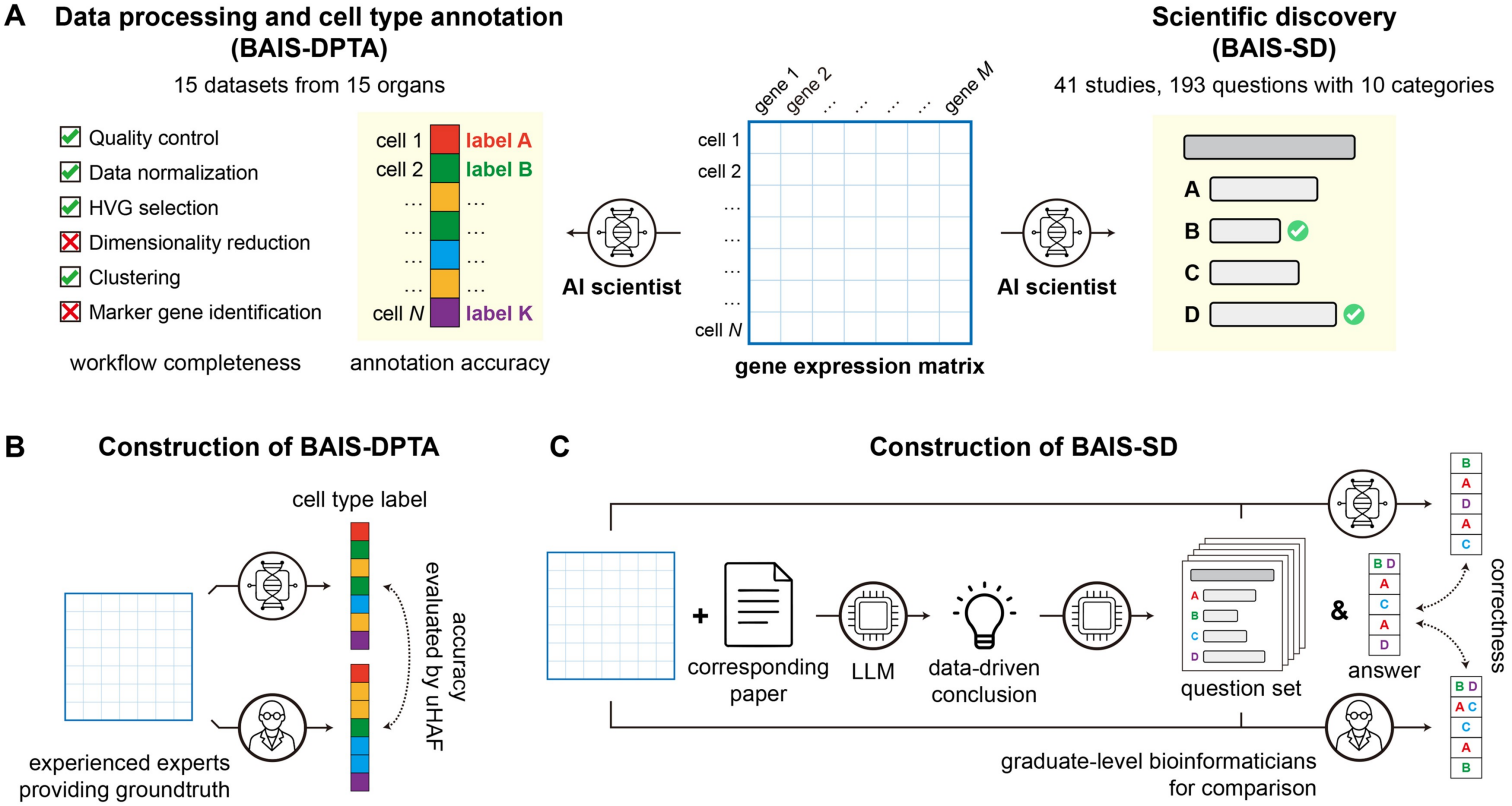
(A) Overview of BAISBench. (B) Construction of BAIS-DPTA: multi-organ single-cell transcriptomic datasets were curated and expert-annotated to support hierarchical cell type evaluation using uHAF. (C) Construction of BAIS-SD: published single-cell studies and corresponding datasets were curated to derive data-driven multiple-choice questions, which were completed by both AI scientists and graduate-level bioinformaticians.

Here, we introduce BAISBench (Biological AI Scientist Benchmark), a benchmark designed to evaluate AI scientists in realistic, data-driven biological discovery settings. BAISBench is built upon single-cell transcriptomic datasets, a representative and widely adopted modality in contemporary molecular and cellular biology. Importantly, scRNA-seq analysis involves multiple stages that require both technical proficiency and domain-specific biological knowledge, making it a suitable testbed for assessing whether AI scientists can integrate data analysis with biological reasoning. BAISBench consists of two complementary tasks (Fig. 1A):

- **Data Processing and cell Type Annotation (BAIS-DPTA)**: This task evaluates fundamental analytical capabilities using 15 expert-labeled single-cell datasets and introduces a hierarchical, cell type–aware metric that captures both annotation accuracy and biological granularity.
- **Scientific Discovery (BAIS-SD)**: This task comprises 193 multiple-choice questions derived from biological conclusions reported in 41 published single-cell studies, requiring AI scientists to analyze corresponding datasets and identify conclusions consistent with established findings.

Using BAISBench, we systematically evaluate several state-of-the-art AI scientists designed for single-cell analysis. To provide human performance baselines, we additionally invited six graduate-level bioinformaticians: one completed the BAIS-DPTA task using an automated cell annotation tool, and five collectively completed the BAIS-SD task. Our results show that while current AI scientists remain less effective than human experts in tasks requiring deep biological judgment, they are already capable of reliably executing standard preprocessing and analytical workflows. Moreover, in the scientific discovery task, the best-performing AI scientists achieve performance comparable to that of graduate-level human researchers, with overall accuracy largely determined by the underlying base LLM models.

Together, these findings position BAISBench as a biologically grounded evaluation framework that characterizes both the capabilities and the limitations of current AI scientists in data-driven biological discovery. By providing a realistic and interpretable benchmark, BAISBench offers a principled basis for assessing progress in AI-assisted biology and for guiding the development and selection of AI systems that can meaningfully support real-world research workflows.

## Methods

To evaluate AI scientists in realistic, data-driven biological research settings, we developed BAISBench, a benchmark composed of two complementary tasks that probe distinct but interrelated capabilities required for biological discovery (Fig. 1A). The first task, Data Processing and Cell Type Annotation (BAIS-DPTA), assesses whether an AI scientist can reliably execute standard single-cell analytical workflows and apply biological knowledge to identify cell identities from gene expression profiles, without relying on external automated annotation tools such as CellTypist. The second task, Scientific Discovery (BAIS-SD), evaluates higher-level biological reasoning by requiring AI scientists to analyze real single-cell datasets and answer questions derived from published biological discoveries. Together, these two tasks are designed to decompose biological research into foundational analytical competence and integrative discovery-oriented reasoning, allowing systematic assessment of current AI scientists. The design and construction of both tasks are described in detail below.

### The data processing and cell type annotation (BAIS-DPTA) task

The BAIS-DPTA task targets the most fundamental layer of single-cell data analysis, encompassing both computational execution and biologically informed interpretation. While many preprocessing steps can be standardized, accurate cell type annotation remains highly dependent on domain-specific biological knowledge and contextual judgment, making this task well suited for benchmarking core analytical capabilities of AI scientists.

As illustrated in Fig. 1B, we curated 15 scRNA-seq datasets from recent publications, each representing a distinct organ or tissue. These datasets were carefully annotated by multiple experts during the human Ensemble Cell Atlas (hECA) project (Chen et al. 2022, Xi et al. 2025), ensuring the accuracy of the cell type label. All datasets were selected to cover a broad range of tissue types, cell numbers (2312–58 706), and annotation granularities (4–42 cell types). Detailed information on the source studies and datasets is provided in Table 3, available as supplementary data at *Bioinformatics* online. All datasets are provided in standardized h5ad format with raw gene expression counts.

To establish high-quality reference annotations, a team of experienced bioinformaticians manually annotated cell types in each dataset using the hierarchical taxonomy defined by the Unified Hierarchical cell Annotation Framework (uHAF) (Bian et al. 2025). uHAF provides organ-specific hierarchical cell type trees (uHAF-Ts) constructed from anatomical literature, single-cell studies, and the Cell Ontology, covering 50 organs. This hierarchical structure enables cell type annotations to be compared at multiple levels of biological granularity and supports systematic evaluation beyond exact label matching.

**Figure 2:**
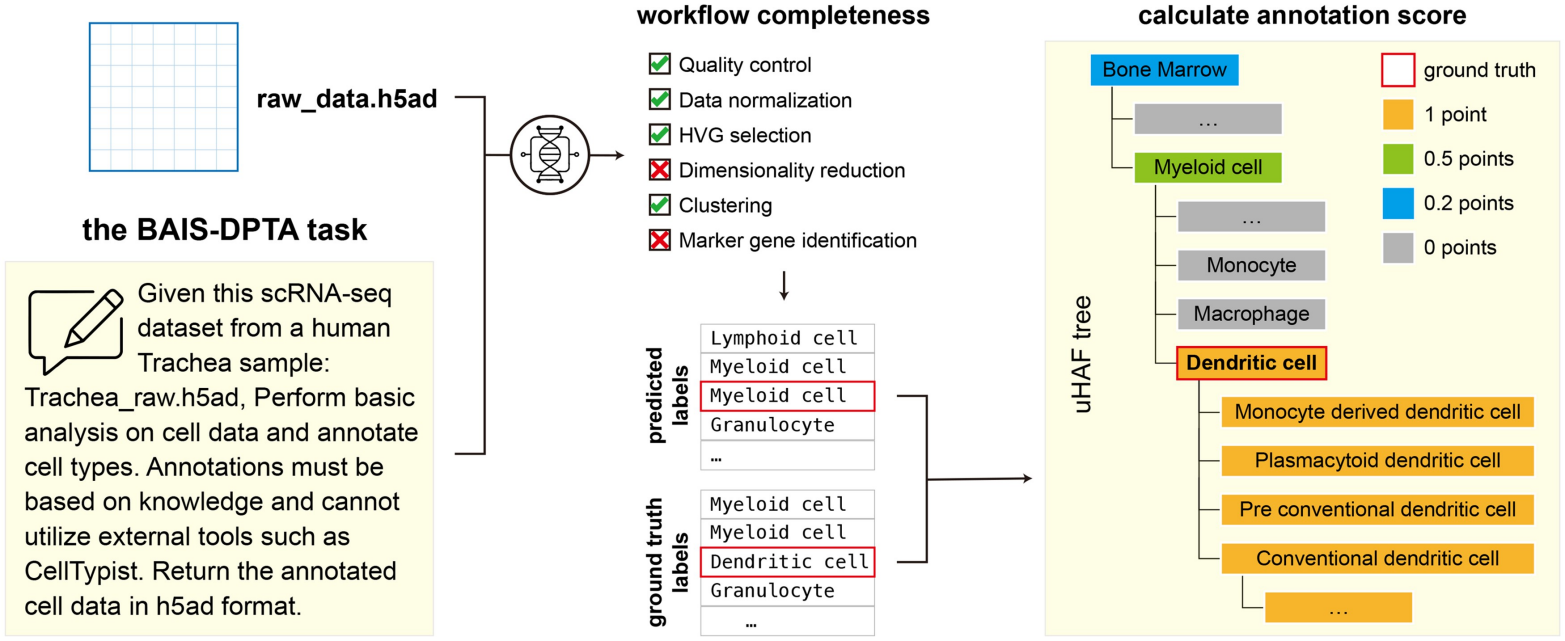
Pipeline of the BAIS-DPTA task. The AI scientist is provided with a single-cell gene expression dataset from a specific organ and is required to perform cell type annotation using its own chosen method. The predicted annotations are then evaluated using a hierarchical scoring metric based on the uHAF cell type tree, which quantifies performance according to the granularity and correctness of the predictions.

In BAIS-DPTA, each AI scientist is provided with a single dataset and is free to adopt any analysis strategy for preprocessing and cell type annotation (Fig. 2). After completing the analysis, we first assess whether the AI scientist executed a complete and biologically reasonable preprocessing workflow. Specifically, we examine the presence of six essential steps commonly required in single-cell analysis: quality control, data normalization, highly variable gene (HVG) selection, dimensionality reduction, clustering, and marker gene identification.

We then evaluate cell type annotation performance using a uHAF-based hierarchical score $S_{\text{CTA}}$ that assigns full, partial, or weak credit to exact (or finer), parent, and grandparent (or equivalent higher-level ancestor)-level matches, respectively:


(1) $$S_{\text{CTA}}=\frac{n_{\text{exact}}+0.5n_{\text{parent}}+0.2n_{\text{grand}}}{n_{\text{cell}}}$$

where $n_{\text{cell}}$ is the total number of cells. This metric explicitly rewards biologically meaningful partial correctness and reflects the hierarchical nature of cell type definitions.

### The scientific discovery (BAIS-SD) task

For the BAIS-SD task, we constructed a set of multiple-choice questions derived from the biological discoveries reported in recently published single-cell research papers. This task is designed to evaluate the ability of AI scientists to generate biologically meaningful insights in realistic research scenarios. It requires not only effective data processing and analysis, but also appropriate tool usage, substantial domain-specific knowledge, and strong reasoning capabilities.

We curated 41 recently published single-cell studies together with their corresponding datasets from the CellxGene platform (Program et al. 2025, Abdulla et al. 2025). These datasets span a wide range of biological contexts and contain between 5600 and 122 000 cells, along with metadata necessary for downstream analyses. A complete list of the studies and datasets is provided in Table 4, available as supplementary data at *Bioinformatics* online.

From each study, we constructed multiple-choice questions grounded in key biological findings reported in the original publication (Fig. 1C). To ensure consistency and scalability, we used an LLM-assisted procedure to extract candidate discoveries and convert them into questions. Specifically, we provided each paper to GPT-4o and instructed it to:

1. Give a quick and short summary of the research background in the first person, and extract the basic information about the sequencing data;
2. Identify which conclusions/discoveries in the article are derived directly from the single-cell transcriptomic data measured by the authors, and list them all;
3. Identify which conclusions/discoveries in the article are based on a combination of data measured by the author and external knowledge, and list them all;
4. Select five appropriate conclusions/discoveries from the above and formulate them into multiple-choice questions.

The full prompt is provided in Supplementary Materials.

**Figure 3:**
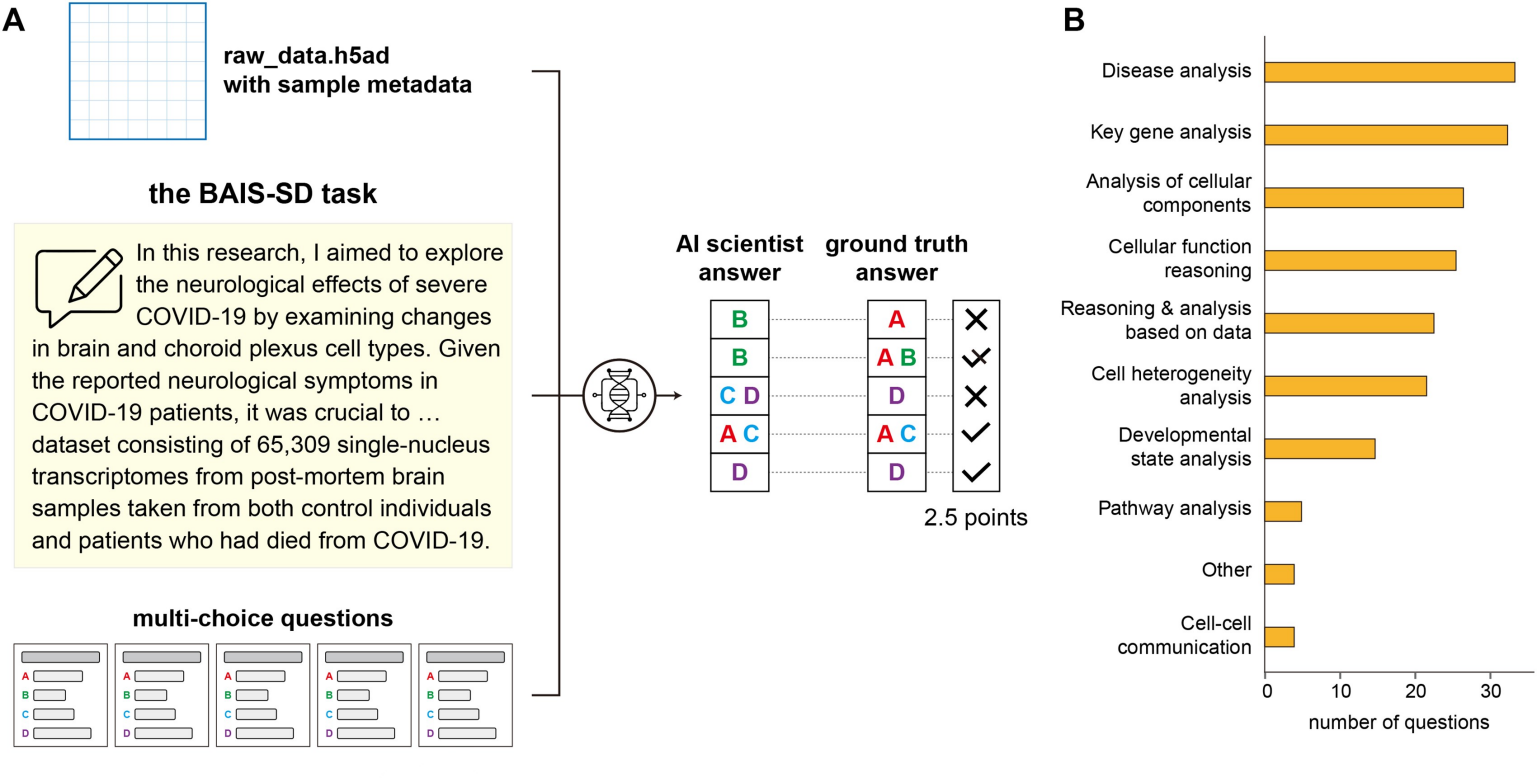
(A) Pipeline of the BAIS-SD task. The AI scientist is provided with background information and a corresponding single-cell dataset, and is required to answer multiple-choice questions by performing data analysis and biological reasoning. Its answers are then compared against the ground truth derived from published literature. (B) Distribution of question categories in the BAIS-SD multiple-choice question set.

To ensure the accuracy and feasibility of the questions, we invited a human expert (details shown in Table S1, available as supplementary data at *Bioinformatics* online) to review and revise all questions generated by the LLM. During this process, we ensured that each question accurately reflected the biological conclusions reported in the original studies and could be solved by analyzing the corresponding data. The final BAIS-SD benchmark contains 193 questions, of which approximately 20% are multiple-answer questions. As shown in Fig. 3B, questions span diverse analytical themes, including key gene analysis, cellular heterogeneity, functional interpretation, and developmental or disease-related reasoning. By grounding questions in published discoveries, BAIS-SD emphasizes the generation of interpretable biological understanding rather than the reproduction of specific computational outputs.

**Table 1:** Workflow completeness of different AI scientists on the BAIS-DPTA task. Data norm. denotes data normalization, dim. reduction denotes dimensionality reduction, and # marker genes denotes the number of marker genes used for cell type annotation.

	Human expert	Human w/CellTypist	GPT-4o	AutoBA	scChat	Biomni	Pantheon	STELLA
**Quality control**	√	√	×	√	√	√	√	√
**Data norm.**	√	√	√	√	√	√	√	√
**HVG selection**	√	√	√	√	√	√	√	√
**Dim. reduction**	√	√	√	√	√	√	√	√
**Clustering**	√	–	√	√	√	√	√	√
**# marker genes**	1∼3	–	3	2∼3	1∼30	5∼8	3	3∼4

As illustrated in Fig. 3B, the questions in BAIS-SD are organized into several major categories. Some questions correspond to conventional analytical tasks commonly encountered in single-cell studies, such as key gene analysis and cell heterogeneity analysis. In parallel, BAIS-SD also includes questions that probe discovery-oriented biological reasoning, such as cellular function reasoning and developmental state analysis. By jointly incorporating both established analytical tasks and questions derived from published biological discoveries, BAISBench goes beyond evaluating computational proficiency alone and directly assesses whether AI scientists can generate biologically meaningful and interpretable insights from data.

Several example questions are presented below to illustrate the purpose and scope of this task:

Examples of multi-choice questions **What role did FOXL1+ fibroblasts play in intestinal development, as observed in the data?** 

- They were involved in crypt-villus differentiation by expressing BMP ligands.
- They inhibited the differentiation of intestinal stem cells in the fetal gut.
- They acted as the major source of WNT3A in the developing intestine.
- They were the primary source of VEGF for endothelial cell development.

**Based on a combination of transcriptomic data and external knowledge, which of the following factors are proposed to contribute to increased proliferation in foreskin keratinocytes?** 

- High expression of amphiregulin (AREG)
- Increased activation of TGF-β signaling
- Low levels of CXCL14 and CCL27
- Presence of CD1C+CD301A+ myeloid dendritic cells

In the BAIS-SD task, each AI scientist is provided with a dataset and minimal background context and is required to answer five questions by performing data analysis and biological reasoning (Fig. 3A). The AI scientists must preprocess the data, conduct relevant analyses using appropriate tools, and reason through the question in order to select the correct options. Performance is quantified using an accuracy-based score. For single-answer questions, one point is awarded for a correct answer. For multiple-answer questions, one point is awarded if all correct options are selected, 0.5 points if at least one correct option is selected and no incorrect options are chosen, and zero otherwise. The overall scientific discovery score $S_{\text{SD}}$ is defined as:


(2) $$S_{\text{SD}}=\frac{n_{\text{single}}+n_{\text{multi-all}}+0.5n_{\text{multi-part}}}{n_{Q}}\times 100$$

where $n_{Q}$ is the total number of questions.

### Evaluated AI scientists

To comprehensively assess the current landscape of AI scientists for biological research and characterize how different system designs affect performance on BAISBench, we evaluated five representative AI scientists spanning a broad range of architectural and methodological choices: AutoBA (Zhou et al. 2024), scChat (Lu et al. 2024b), Biomni (Huang et al. 2025), Pantheon (pan, 2025), and STELLA (Jin et al. 2025).

The details of each AI scientist can be found in the supplementary material. These systems span a broad design space, ranging from pipeline automation and interactive analysis to structured multi-agent reasoning and hybrid model integration. Evaluating them side by side enables systematic analysis of how architectural choices influence AI scientists’ ability to support data-driven biological discovery.

## Results

### The performance of AI scientists on the BAIS-DPTA task

The BAIS-DPTA task evaluates whether AI scientists can reliably execute standard single-cell analytical workflows and apply biological knowledge to cell type annotation. To enable broader participation and provide an additional reference point, we included a baseline in which GPT-4o directly generated executable Python code for data preprocessing and cell type classification. As a human reference, a graduate-level bioinformatician completed the same task using an automated annotation tool CellTypist (Domínguez Conde et al. 2022) following its official workflow.

#### Evaluation of basic data processing and analysis workflows

As summarized in Table 1, all evaluated AI scientists were able to complete the full preprocessing and analysis pipeline, successfully executing all six required steps: quality control, data normalization, HVG selection, dimensionality reduction, clustering, and marker gene identification. This indicates that current AI scientists have largely mastered the procedural aspects of standard single-cell analysis workflows.

Despite this overall consistency, we observed substantial methodological variation across systems. GPT-4o selected overly stringent quality-control thresholds, resulting in excessive filtering of cells and genes; this issue was manually corrected to ensure fair comparison. Only Biomni, Pantheon, and STELLA performed explicit data scaling prior to downstream analysis. scChat adopted the scVI model for dimensionality reduction, whereas other systems relied on principal component analysis (PCA). For clustering, Biomni explored multiple resolution parameters, while Pantheon and STELLA uniquely employed the scipy implementation of *k*-means clustering; all other systems used the Leiden algorithm implemented in Scanpy. Marker gene selection strategies also differed, with most systems selecting three marker genes per cell type, while Biomni selected a broader set of five to eight genes.

Furthermore, we observe distinct patterns of success across AI scientist architectures at each processing step. Systems with hard-coded toolkits and predefined workflows (e.g. AutoBA and scChat) exhibit all-or-nothing behavior, where success or failure on a single dataset generalizes across all datasets, such as AutoBA failing at the final cell type labeling step. In contrast, more flexible multi-agent systems such as Biomni incorporate verification loops and adaptive strategies, allowing them to consistently complete each step, albeit not always optimally, resulting in higher overall success rates.

Together, these results show that contemporary AI scientists are generally capable of autonomously executing complete and technically sound single-cell preprocessing and analysis pipelines, even though their specific methodological choices vary substantially.

#### Cell type annotation performance

**Figure 4:**
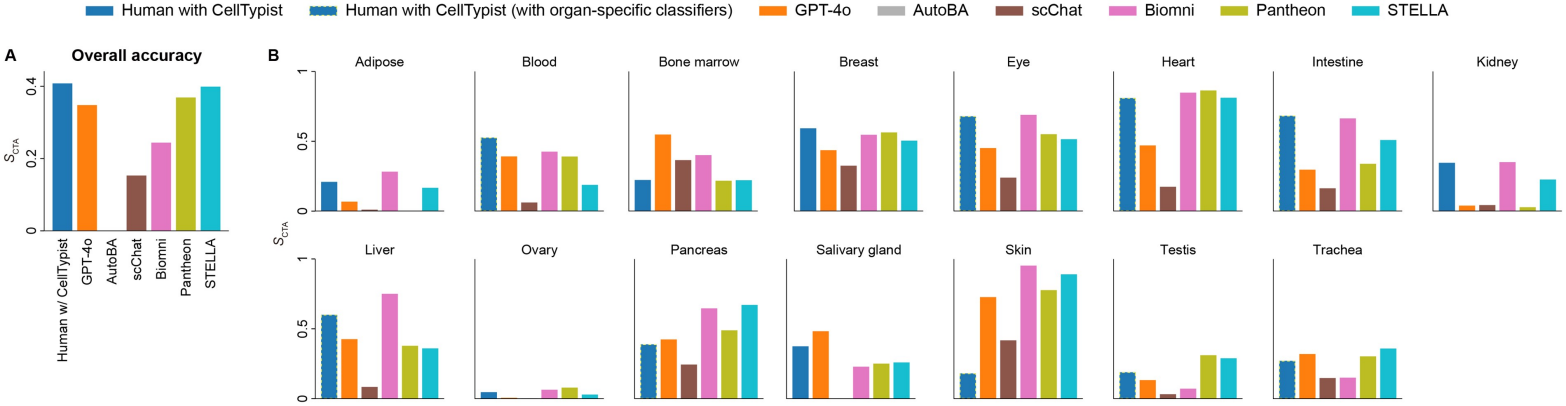
The cell type annotation accuracy of different AI models in the BAIS-DPTA task. (A) Overall results. (B) Results on different organs. The results of AutoBA are not shown in (B) as they are all zero.

In contrast to the uniformly strong performance in workflow execution, we observed greater divergence in cell type annotation accuracy. As shown in Fig. 4A, the human expert using CellTypist achieved the highest overall accuracy. Among the AI scientists, STELLA achieved the best performance, although it remained slightly below the human–CellTypist baseline. AutoBA failed to complete the annotation task, as it was unable to assign cell labels after identifying marker genes.

Notably, several AI scientists, including AutoBA, scChat, and Biomni, performed worse than the baseline approach in which GPT-4o directly generated executable code for cell type classification. This result suggests that increased system complexity or architectural sophistication does not necessarily translate into improved biological interpretation when explicit domain knowledge must be applied.

Analysis across individual organs revealed broadly consistent performance trends (Fig. 4B). Most methods performed well on certain tissues, such as heart, but struggled on others, including ovary. Notably, CellTypist provides both organ-specific classifiers trained on dedicated tissue datasets and more general-purpose models trained on broader immune or cross-tissue data. Although organ-specific classifiers are expected to achieve superior annotation accuracy on their corresponding tissues, we found that they did not consistently outperform AI scientists across multiple organs. This observation suggests that AI scientists may possess the capacity to adapt their analysis and incorporate organ-specific knowledge in a data-driven manner, highlighting their potential as flexible alternatives to traditional automated annotation tools.

We further examined the annotation strategies adopted by different systems. Pantheon and STELLA assigned cell types by computing module-level scores using the Scanpy score_genes function and selecting labels based on these scores, whereas other systems primarily relied on overlap between differentially expressed genes and predefined marker genes. These strategy differences were associated with measurable performance differences.

**Figure 5:**
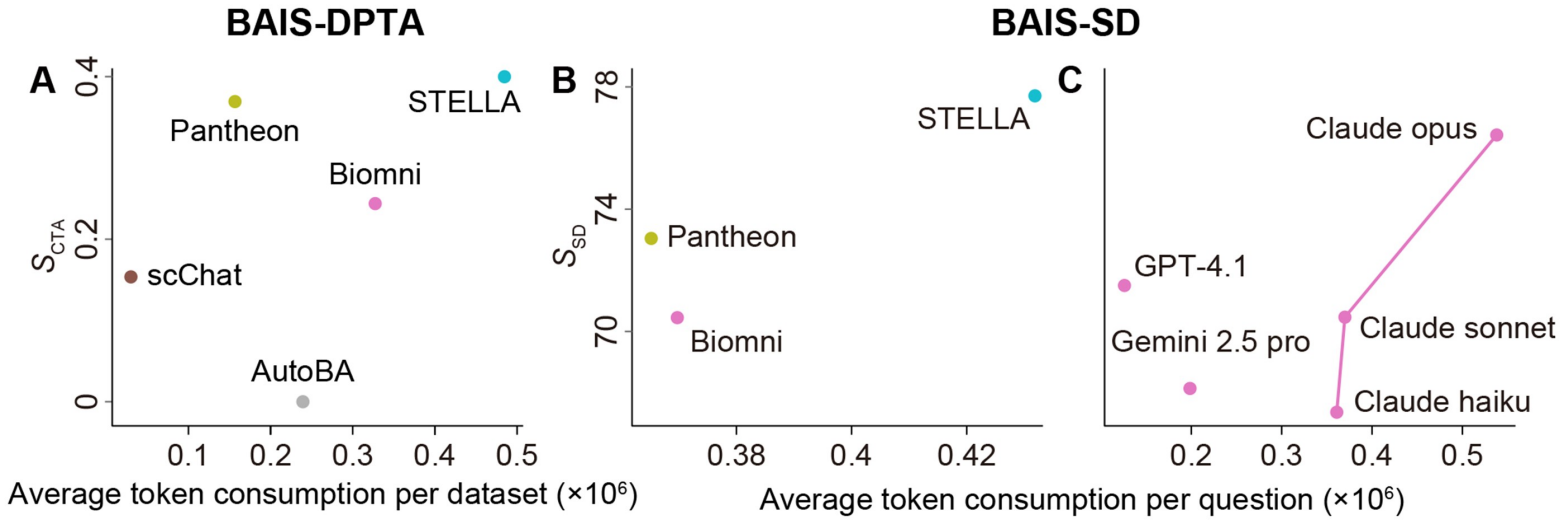
The relationship of AI scientist performance and LLM token consumption in (A) BAIS-DPTA, (B) BAIS-SD using different AI scientists, and (C) BAIS-SD using Biomni with different base LLM models.

Finally, we analyzed the relationship between annotation performance and total LLM token consumption (Fig. 5A). Excluding AutoBA, which failed to produce valid annotations, we observed a clear positive correlation between performance and token usage across AI scientists. This trend suggests that, under the current paradigm, improved performance in biologically informed annotation is achieved largely through increased computational and reasoning resources.

#### Summary of BAIS-DPTA results

Taken together, these results reveal a clear capability gap in current AI scientists. While they are largely proficient at executing standardized single-cell preprocessing and analysis workflows, they remain less reliable than human experts for tasks that require nuanced biological judgment, such as accurate cell type interpretation. At the same time, although CellTypist guided by a human expert achieves the highest overall accuracy, AI scientists outperform it on specific organs, highlighting their potential to complement and, in some cases, surpass human-guided annotation workflows.

### The performance of AI scientists on the BAIS-SD task

The BAIS-SD task evaluates whether AI scientists can generate biologically meaningful insights from real single-cell datasets by answering questions derived from published discoveries. Unlike BAIS-DPTA, this task requires not only correct execution of data analysis pipelines, but also integrative reasoning that combines experimental evidence with biological knowledge.

Not all evaluated AI scientists were able to complete the BAIS-SD task. scChat was excluded because its function tools are hard-coded and do not support the customized analytical workflows required for this benchmark. AutoBA was also excluded, as it consistently failed to produce valid answers. Consequently, our evaluation focused on three AI scientists: Biomni, Pantheon, and STELLA. Biomni and Pantheon both adopted Claude Sonnet 4.5 as their underlying base LLM model, whereas STELLA employed a heterogeneous configuration that combines Claude Sonnet 4.5, Gemini 2.5 Pro, and Grok-4 to handle different subtasks. Each AI scientist ran the task for 10 times to ensure the stability and reliability of results.

To provide a human performance reference, we additionally invited five graduate-level bioinformaticians to independently complete the same question set. Each participant was randomly assigned one-fifth of the questions, collectively covering the full BAIS-SD benchmark. Detailed information of these participants is shown in Table S1, available as supplementary data at *Bioinformatics* online.

#### Multiple-choice question answering performance

**Figure 6:**
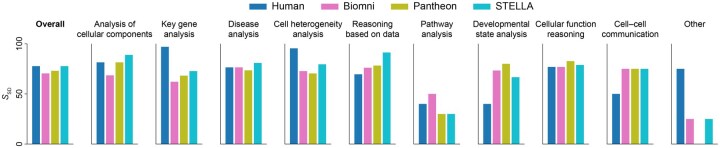
The performance of AI scientists on the BAIS-SD task.

Overall performance on the BAIS-SD task is summarized in Fig. 6. Among the evaluated AI scientists, STELLA achieved the highest overall score, reaching performance comparable to that of human experts. Pantheon ranked second, followed by Biomni. This result indicates that, under appropriate system designs, AI scientists can already approach human-level performance in data-driven biological discovery tasks. The results are also consistent across different runs (Table 2, available as supplementary data at *Bioinformatics* online), likely due to the use of predefined analytical workflows and standardized processing pipelines, which reduce variability in problem-solving strategies. In addition, built-in feedback mechanisms help ensure that analyses are carried out to a satisfactory level.

Two factors likely contribute to STELLA’s superior performance. First, its modular design allows different subtasks to be handled by base LLM models best suited to their respective requirements, facilitating more effective integration of data analysis and biological reasoning. Second, STELLA consumed a substantially larger number of tokens than the other systems (Fig. 5B), enabling more exhaustive exploration of the data and reasoning space. Together, these observations suggest that both architectural flexibility and computational budget play important roles in achieving high performance on discovery-oriented tasks.

#### Performance across biological question categories

To better understand model behavior, we further examined performance across ten question categories (Fig. 6). Despite differences in absolute accuracy, all three AI scientists exhibited similar performance trends, with certain question types consistently easier or more challenging across systems. Notably, STELLA outperformed the other AI scientists in nearly all categories, indicating robust advantages across diverse types of biological reasoning.

Human experts showed clear advantages in key gene analysis and cell heterogeneity analysis, reflecting the importance of domain expertise and interpretative judgment in these tasks. In contrast, AI scientists often matched or exceeded human performance in data-driven reasoning and developmental state analysis, where systematic and exhaustive exploration of high-dimensional data may confer an advantage. These results suggest that AI scientists and human experts exhibit complementary strengths across different aspects of biological discovery.

Taken together, these findings indicate that current AI scientists are already capable of achieving human-comparable performance on several discovery-oriented tasks. Although a gap to the ideal performance ceiling remains, particularly for tasks requiring nuanced biological interpretation, the observed results highlight substantial potential for AI scientists in supporting data-driven biological research.

#### Impact of base LLM models on BAIS-SD performance

To disentangle the effects of system architecture and underlying base LLM models, we further evaluated the Biomni framework using different LLM models. Specifically, we tested Claude Haiku 3.5 as a weaker baseline, Claude Opus 4.5 as a stronger baseline, and two models from other providers, GPT-4.1 and Gemini 2.5 Pro.

As shown in Fig. 5C, the choice of base LLM model had a pronounced impact on BAIS-SD performance. Within the Claude family, performance followed a clear ordering of Opus > Sonnet > Haiku, with Claude Opus 4.5 elevating Biomni’s performance to a level close to that of human experts. One contributing factor is that stronger models typically operate with higher token budgets, and within the same architecture, AI scientist performance was positively correlated with token usage.

Interestingly, GPT-4.1 and Gemini 2.5 Pro achieved performance comparable to Claude Sonnet 4.5 while requiring fewer tokens (Fig. 5C), suggesting differences in efficiency across base LLM models. Overall, these results indicate that, relative to system architecture, the choice of base LLM models plays a more decisive role in determining performance on BAIS-SD. Stronger base models can directly and substantially enhance AI scientists’ ability to generate biologically meaningful insights from data.

#### Check for potential “cheating” behavior

A key concern in evaluating AI scientists is whether performance reflects genuine data-driven reasoning or shortcut behaviors such as memorization or retrieval. To investigate this, we first evaluated BAIS-SD without providing datasets. Although performance decreased, accuracy remained well above random (Fig. 1, available as supplementary data at *Bioinformatics* online), suggesting that systems can partially answer questions without analyzing data.

This behavior is largely due to the removal of the prompt constraint requiring answers to be derived from the provided data. Without this constraint, systems can leverage existing knowledge bases and external retrieval to obtain the answer, particularly since questions are derived from published studies.

To distinguish shortcut-based answering from data-driven reasoning, we conducted a controlled experiment by randomly shuffling datasets and pairing each question with an unrelated dataset, while preserving the original constraint. If performance relies on memorization or retrieval, accuracy should remain stable; otherwise, it should decline.

As shown in Fig. 2, available as supplementary data at *Bioinformatics* online, performance drops substantially under mismatched data, and systems frequently report insufficient information. These results indicate that the prompt constraint effectively enforces data-dependent reasoning and limits shortcut behaviors, supporting the validity of BAISBench as a data-driven evaluation framework. More broadly, these findings suggest that appropriately designed constraints are essential for preventing shortcut behaviors.

#### Summary of BAIS-SD results

Taken together, the BAIS-SD results demonstrate that current AI scientists are already capable of performing several discovery-oriented tasks at a level comparable to human experts. While human researchers retain clear advantages in tasks that rely heavily on domain intuition and interpretative judgment, such as key gene identification and cell heterogeneity analysis, AI scientists often match or exceed human performance in data-driven reasoning and developmental state analysis. These complementary strengths suggest that AI scientists are particularly effective when systematic exploration of high-dimensional data is required.

At the same time, performance differences across systems and base LLM models indicate that current capabilities remain strongly constrained by the underlying language models and available computational resources. Together, these findings suggest that AI scientists have begun to cross a threshold from purely assistive tools toward meaningful participants in data-driven biological discovery, while still falling short of fully autonomous scientific reasoning.

## Discussion

In this work, we introduce BAISBench as a data-driven benchmark for evaluating AI scientists in realistic biological research scenarios. BAISBench decomposes biological research into two complementary tasks, BAIS-DPTA and BAIS-SD, with the goal of assessing both procedural analytical competence and data-driven biological reasoning. This design reflects that AI scientists comprise multiple heterogeneous capabilities rather than a single unified skill.

The results demonstrate that evaluating AI scientists is fundamentally different from evaluating standalone models. AI scientists operate as agentic systems that must plan, execute, and iteratively refine analyses over extended trajectories. As a result, failures often arise from system-level behaviors such as brittle execution, error accumulation, and mismatches between planning and implementation. BAISBench is designed to surface these behaviors by grounding evaluation in end-to-end tasks built on real single-cell datasets.

Despite encouraging progress, several limitations remain. Many systems can generate plausible high-level plans but struggle to translate them into robust, executable code, particularly in multi-step workflows where small errors can propagate. In addition, dependence on rigid pipelines limits generalization, and the integration of external biological knowledge remains limited. These observations suggest that current AI scientists are more reliable as assistants than fully autonomous agents.

BAISBench explicitly incorporates human baselines to anchor performance in realistic research practice. However, human evaluation remains limited in scale, as tasks were distributed across participants and inter-annotator variability was not systematically assessed. Future work could include inviting more human participants to complete the BAIS-DPTA task using different approaches to establish a human performance baseline, or inviting more participants to complete the BAIS-SD task to assess the agreement among human participants on this task and provide a more convincing baseline.

It is also important to clarify the scope of what BAISBench measures. For the BAIS-SD task, the benchmark evaluates whether systems can recover established biological conclusions from data rather than generate novel discoveries. Given the inherent ambiguity of biological analysis, imperfect scores reflect deviations from evidence-based interpretations reported in the literature rather than absolute failure. In addition, BAISBench currently focuses primarily on biological analysis driven by scRNA-seq; however, there are many other types of biological data in the field of biology. Future directions will include designing questions that encompass a wider range of biological modalities to provide more comprehensive coverage of various aspects of the biological field.

Taken together, these observations point toward a near-term trajectory in which AI scientists are rapidly evolving from human assistants to autonomous AI researchers. BAISBench serves as a diagnostic framework to identify system limitations, benchmark progress, and support the development of more reliable and biologically grounded AI scientists.

## Supplementary Material

btag227_Supplementary_Data

## Data Availability

The data used in this manuscript can be found at: https://huggingface.co/datasets/EperLuo/BaisBench.
