# Supplementary material for: Benchmarking AI scientists for omics data–driven biological discovery
Source: Bioinformatics. 2026 Jul 7;42(Suppl 1):btag227. doi: 10.1093/bioinformatics/btag227 (PMC13340177; doi:10.1093/bioinformatics/btag227)
Supplement: btag227_Supplementary_Data [file btag227_supplementary_data.zip › Zhang.105.sup.1.pdf]

Supplementary Materials

Information of Evaluated AI scientists

AutoBA

AutoBA is designed as a lightweight, automation-oriented AI scientist that focuses on constructing end-to-end pipelines for single-cell data preprocessing and analysis. It primarily emphasizes tool orchestration and code generation to execute standard analytical steps with minimal human intervention.

scChat

scChat adopts an interactive, conversation-driven paradigm for single-cell data analysis. By combining a multi-agent architecture with retrieval-augmented generation, scChat enables users to explore datasets through natural language queries while dynamically invoking analytical tools. This design makes scChat particularly suitable for exploratory analysis and human–AI interaction.

Biomni

Biomni is a general-purpose AI scientist framework tailored for biomedical research. It integrates literature retrieval, tool-assisted data analysis, and multi-step reasoning within a unified system, allowing it to combine experimental data with external biological knowledge. Biomni is designed to support autonomous biomedical workflows beyond individual tasks, making it more flexible than pipeline-centric systems.

Pantheon

Pantheon emphasizes structured scientific reasoning through coordinated multi-agent collaboration. It decomposes complex research objectives into iterative planning, execution, and evaluation steps, enabling systematic exploration of biological hypotheses. Compared to more tool-centric systems, Pantheon places greater emphasis on reasoning consistency and decision-making structure, which can be advantageous for multi-step analytical tasks.

STELLA

STELLA represents a hybrid approach that tightly couples LLMs with tool-assisted data analysis and task-specific agent roles. By leveraging multiple base LLM models for different subtasks, STELLA is designed to balance analytical accuracy, reasoning depth, and robustness across diverse biological scenarios. This modular design allows STELLA to adapt to different stages of the biological research workflow, making it particularly well-suited for complex discovery-oriented tasks that require both data analysis and biological interpretation.

Details of prompts

Prompt for AI scientist in the BAIS-DPTA

Given this scRNA-seq dataset from a human Adipose sample: $/path/to/Adipose_raw.h5ad$, Perform basic analysis on cell data and annotate cell types. Annotations must be based on knowledge and cannot utilize external tools such as CellTypist. Return the annotated cell data in h5ad format. Save the annotated file in $/path/to/Adipose.h5ad$

Prompt for GPT-4o in the BAIS-DPTA

Given a scRNA-seq dataset from a human intestine sample (in h5ad format). Write code to perform basic analysis on cell data and annotate cell types. Annotations must be based on knowledge and cannot utilize external tools such as CellTypist.

Prompt for constructing multi-choice questions

I need to assign an assignment for a bioinformatics analysis class that is about giving a single cell transcriptome data and answering questions by analyzing the given data. I now need to design the questions based on what is in the original article that corresponds to this dataset. I will give you the article below and ask you to read the contents of this article carefully and complete the following tasks:

1. A quick and short summary of the research background in the first person. And the basic information about the sequencing data.

2. Consider which of the conclusions/discoveries in the article are derived directly from the single-cell transcriptome data measured by the authors? Please list them all (use ”the data ...” instead of ”the study ...”, ”the author ...” or ”the research ...”); this will serve as fodder for my questions. Be as specific as possible, include specific key terms or descriptions, and if necessary, include the process or intermediate steps that led to the conclusion, no generalizations, no descriptive words.

3. Consider which conclusions/discoveries in the article are based on a combination of data measured by the author and external knowledge. Please list them all, this is also my source material for the questions, all requirements are strictly the same as the one above.

4. Choose 5 appropriate conclusions/discoveries from the above and form them into multiple-choice questions (each entry is a separate question). It can be either single (only one correct answer, like ”B”) or multi-answer (more than one correct answer, like ”ACD”) questions. Make the position of the correct answer as random as possible. The correct option comes from the article; the incorrect option can come from the article or be added from your own knowledge, but not judged too easily. Give the correct answer to the questions. I would not give the article to the students, so don’t come up with anything that need to reading the article That is, avoid expressions like “xxx in the study”, ”the author ...” or ”xxx in the research”, instead, using ”in the data ...”. What you need to do is treat what is in the article as a standard answer so that students can reproduce those conclusions or discoveries from the given transcriptomic data (not any other data).

Example prompt for AI scientist in BAIS-SD

I am a bioinformatician, and I am doing research on newly measured single-cell transcriptomic data. I now give you the data file (path/to/the/data) and the background information about the dataset and my research. I need you to analyze the data to answer the following multiple-choice questions. You can only arrive at the answer by analyzing the data yourself. You can only use your existing knowledge; you can not search the internet for answers; you cannot directly consult the original literature corresponding to the dataset. Here is the information and the question:

Background:
Lung development is a highly complex process involving a diverse array of cell types, yet our understanding of late-stage human lung development remains incomplete. Animal models have provided critical insights, but translating these findings to human biology is challenging due to species differences. To address this gap, we used single-cell RNA sequencing (scRNA-seq) to create a molecular atlas of newborn human lung cells. This allows us to define distinct cellular populations and their gene signatures, offering new insights into the structural and functional maturation of the human lung at birth. Sample Source: Two one-day-old newborn human lungs were obtained through organ donation. One was from a full-term infant (38 weeks gestational age), and the other was preterm (31 weeks gestational age). Cell Isolation & Processing: Lungs were enzymatically digested to obtain single-cell suspensions, which were frozen and later used for sequencing. Sequencing Platform: Chromium 10X Genomics system (v2 chemistry), sequenced on a HiSeq4000. Final Dataset: 5,499 high-quality cells, including epithelial, endothelial, mesenchymal, and immune cells.

Question:
Which major cell type was found to be the most abundant in the newborn human lung based on single-cell transcriptome data?
A) Endothelial cells
B) Epithelial cells
C) Mesenchymal cells
D) Immune cells

Which of the following markers was specifically associated with immature matrix fibroblasts in the newborn lung?
A) SFTPB
B) HES1
C) CDH5
D) PTPRC

Based on single-cell transcriptomic data, what was a key characteristic of immune cells in the newborn human lung?
A) They were only detected in one of the two donors.
B) They were exclusively macrophages.
C) They included T cells, B cells, and macrophages with donor-to-donor variation.
D) They showed no expression of leukocyte markers.

The estimated developmental state of human newborn lung cells, based on murine postnatal development, was closest to which range of murine postnatal days?
A) 1–3 days
B) 4–9 days
C) 10–15 days
D) 16–20 days

What evidence supports the presence of two distinct matrix fibroblast populations in the newborn lung?
A) Differential expression of EPCAM and PECAM1
B) Separation of cells based on mitochondrial gene content
C) Identification of distinct gene expression profiles, including COL6A3 and TCF21
D) Complete absence of mesenchymal markers in one fibroblast population

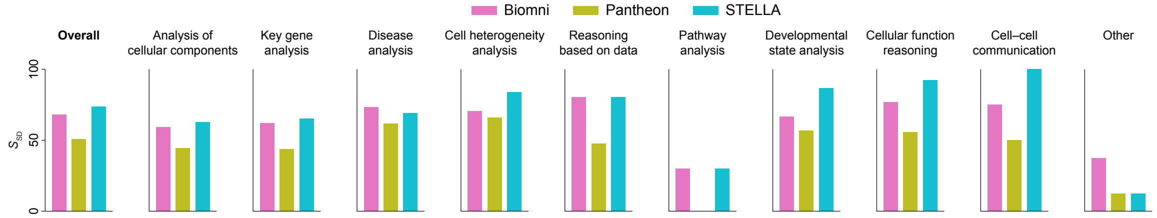

Fig. S1. The performance of AI scientists in BAIS-SD without giving any dataset.

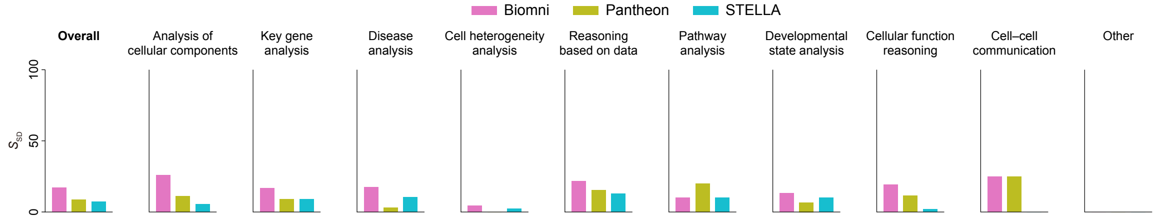

Fig. S2. The performance of AI scientists in BAIS-SD giving an unrelated dataset.

Table S1. The background information of human participants involved in this study.

Label	Role	Background	Level
Reviewer	Review BAIS-SD questions	Bioinformatics, biomedical engineering	Ph.D., Assistant Professor
Participant 1	Answer BAIS-SD questions	Bioinformatics	Master's
Participant 2	Answer BAIS-SD questions	Bioinformatics	Master's student
Participant 3	Answer BAIS-SD questions	Cardiovascular, cardiac surgery	M.D.
Participant 4	Answer BAIS-SD questions	Physiology and pathophysiology	M.D.
Participant 5	Answer BAIS-SD questions	Bioinformatics	Ph.D. student

Table S2. The performance of AI scientists in BAIS-SD under multiple runs

	Biomni	Pantheon	STELLA
Mean	71.10	72.98	77.79
Max	73.05	74.61	79.27
Min	68.13	71.24	76.16
Std	1.57×10^{-2}	1.12×10^{-2}	0.92×10^{-2}

Table S3. Summary of single-cell datasets used in the BAIS-DPTA task.

**Paper**	**Year**	**Journal**	**Organ**	**DOI**	**Cell number**	**Cell type number**
Aizarani et al.	2019	*Nature*	Liver	10.1038/s41586-019-1373-2	9194	12
He et al.	2020	*Genome Biology*	Intestine	10.1186/s13059-020-02210-0	8924	12
Chitiashvili et al.	2020	*Nature Cell Biology*	Ovary	10.1038/s41556-020-00607-4	8561	6
Cao et al.	2020	*Science*	Pancreas	10.1126/science.aba7721	43155	14
Zhao et al.	2020	*Nature Commun*	Testis	10.1038/s41467-020-19414-4	26482	8
Miller et al.	2020	*Developmental Cell*	Trachea	10.1016/j.devcel.2020.01.033	17423	5
Roy et al.	2021	*Cell Reports*	Bone marrow	10.1016/j.celrep.2021.109698	30894	6
Voigt et al.	2021	*Hum Mol Genet*	Eye	10.1093/hmg/ddab140	31870	10
Emont et al.	2022	*Nature*	Adipose	10.1038/s41586-022-04518-2	55150	10
Domínguez Conde et al.	2022	*Science*	Blood	10.1126/science.abl5197	24149	26
Suo et al.	2022	*Science*	Kidney	10.1126/science.abo0510	25955	42
Tabula Sapiens Consortium et al.	2022	*Science*	Breast	10.1126/science.abl4896	11227	13
Tabula Sapiens Consortium et al.	2022	*Science*	Salivary gland	10.1126/science.abl4896	26959	22
Gur et al.	2022	*Cell*	Skin	10.1016/j.cell.2022.03.011	38666	8

Table S4. Summary of single-cell papers and datasets used in the Scientific discovery task.

Paper	Year	Journal	DOI	Cell number
Fan et al.	2019	*Nat Commun*	10.1038/s41467-019-11036-9	20676
Jäkel et al.	2019	*Nature*	10.1038/s41586-019-0903-2	17799
Martin et al.	2019	*Cell*	10.1016/j.cell.2019.08.008	32458
Menon et al.	2019	*Nat Commun*	10.1038/s41467-019-12780-8	20091
Stewart et al.	2019	*Science*	10.1126/science.aat5031	105870
Szabo et al.	2019	*Nat Commun*	10.1038/s41467-019-12464-3	51876
Cowan et al.	2020	*Cell*	10.1016/j.cell.2020.08.013	98348
Elmentaite et al.	2020	*Developmental Cell*	10.1016/j.devcel.2020.11.010	85351
Joseph et al.	2020	*Prostate*	10.1002/pros.24020	122129
Lavaert et al.	2020	*Immunity*	10.1016/j.immuni.2020.03.019	71732
Lee et al.	2020	*Sci Immunol*	10.1126/sciimmunol.abd1554	59572
Lukassen et al.	2020	*The EMBO Journal*	10.15252/embj.20105114	57229
Solé-Boldo et al.	2020	*Commun Biol*	10.1038/s42003-020-0922-4	15457
Wang et al.	2020	*J Exp Med*	10.1084/jem.20191130	14106
Wu et al.	2020	*The EMBO Journal*	10.15252/embj.2019104063	24271
Xiang et al.	2020	*Front Cardiovasc Med*	10.3389/fcvm.2020.00052	9980
Melms et al.	2021	*Nature*	10.1038/s41586-021-03569-1	116313
Yang et al.	2021	*Nature*	10.1038/s41586-021-03710-0	65309
Burclaff et al.	2022	*Cell Mol Gastroenterol Hepatol*	10.1016/j.jcmgh.2022.02.007	12590
Fasolino et al.	2022	*Nat Metab*	10.1038/s42255-022-00531-x	69645
Knight-Schrijver et al.	2022	*Nat Cardiovasc Res*	10.1038/s44161-022-00183-w	60668
Lengyel et al.	2022	*Cell Reports*	10.1016/j.celrep.2022.111838	86708
Opasawatchai et al.	2022	*Front Dent Med*	10.3389/fdmed.2021.806294	6560
Watanabe et al.	2022	*Am J Respir Cell Mol Biol*	10.1165/rcmb.2021-0555OC	57918
Xu et al.	2022	*Sci Rep*	10.1038/s41598-022-17832-6	36908
Horeth et al.	2023	*J Dent Res*	10.1177/00220345221147908	15684
Kurkalang et al.	2023	*Cancer Science*	10.1111/cas.15979	28186
Rustam et al.	2023	*Am J Respir Crit Care Med*	10.1164/rccm.202207-1384OC	115788
Strati et al.	2023	*Cell Reports Medicine*	10.1016/j.xcrm.2023.101158	92676
Whitfield et al.	2023	*Clinical & Translational Med*	10.1002/ctm2.1356	62599
Wiedemann et al.	2023	*Cell Reports*	10.1016/j.celrep.2023.111994	15243
Bhattacharya et al.	2024	*Genes*	10.3390/genes15030298	5499
Binvignat et al.	2024	*JCI Insight*	10.1172/jci.insight.178499	108717
de Vrij et al.	2024	*Commun Biol*	10.1038/s42003-024-06225-2	30130
Guerrero-Murillo et al.	2024	*bioRxiv*	10.1101/2024.01.23.576878	37100
Heimlich et al.	2024	*Blood Advances*	10.1182/bloodadvances.2023011445	66985
Li et al.	2024	*Cell Stem Cell*	10.1016/j.stem.2023.12.013	67996
Mimpen et al.	2024	*The FASEB Journal*	10.1096/fj.202300601RRR	10533
Moerkens et al.	2024	*Cell Reports*	10.1016/j.celrep.2024.114247	22280
Phan et al.	2024	*Nat Commun*	10.1038/s41467-024-45165-7	98848
Rabadam et al.	2024	*JCI Insight*	10.1172/jci.insight.176963	105827
