# Supplementary figures and images for: Benchmarking AI scientists for omics data–driven biological discovery
Source: Bioinformatics. 2026 Jul 7;42(Suppl 1):btag227. doi: 10.1093/bioinformatics/btag227 (PMC13340177; doi:10.1093/bioinformatics/btag227)

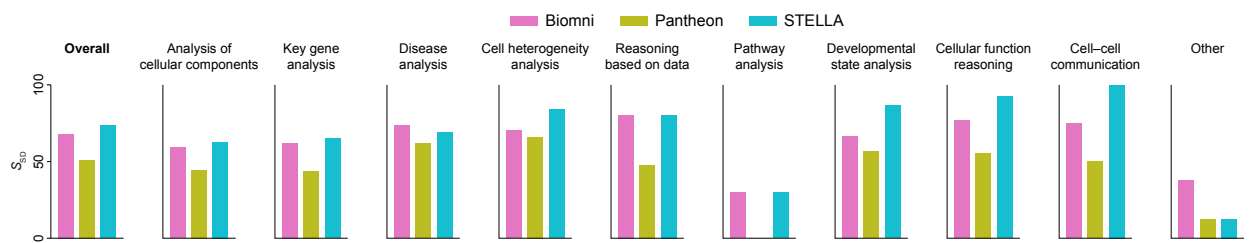

Supplement: btag227_Supplementary_Data [file btag227_supplementary_data.zip › Zhang.105.fig.S1.pdf]

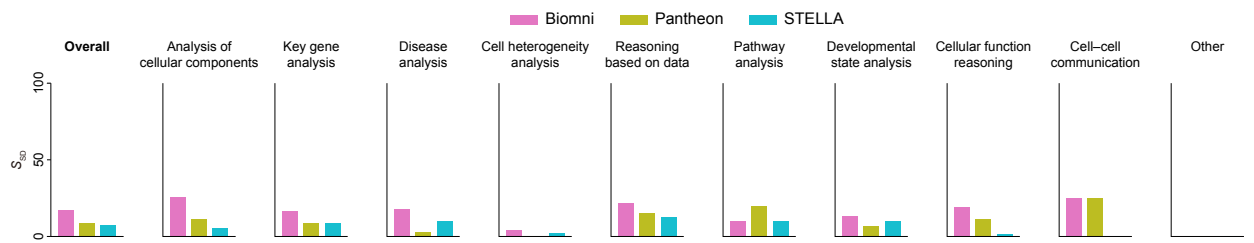

Supplement: btag227_Supplementary_Data [file btag227_supplementary_data.zip › Zhang.105.fig.S2.pdf]
